# Psychosocial and emotional impact of strabismus on Indian families

**DOI:** 10.4103/0301-4738.62665

**Published:** 2010

**Authors:** Parikshit Gogate

**Affiliations:** Dr. Gogate's Eye Clinic, K-102, Kumar Garima, Tadiwala Road, Pune-411 001, India

Dear Editor,

The article ‘Comparison of psychosocial and emotional consequences of childhood strabismus on the families from rural and urban India’ is significant in Indian pediatric ophthalmology.[1] We physicians focus on the ‘disorder’ that we diagnose,[2] rather than on the ‘problem’ that is faced by the patient,[3] which has not just physiological, but also psychological aspects. Strabismus, unfortunately, is not considered a serious ‘disorder’ though most patients with it would call it a ‘significant problem’.

The study, however, focuses on children who were to undergo strabismus surgery.[1] There is an inherent bias, as the parents who considered strabismus to be a ‘problem’ and were ‘distressed’ by it enough would bring their children for the intervention. A more accurate estimation could have been through a population-based study or at least during primary school screening before the children were referred for strabismus surgery. So while the reliability of the questionnaire is high as indicated by the cronbach alpha, perhaps the validity is questionable due to self-selection of the patients and parents. An item response analysis after the surgery would have been more helpful. No doubt the authors shall be presenting these results later.

There is nevertheless a gender bias evident in the study. The cohort of urban paying patients has a male preponderance (11/16, 68.8% are boys), with perhaps girls being neglected. This has been enunciated in studies on role of gender in accessing eye care services.[4] Interestingly, the non-paying rural cohort has more girls (47/77, 61% are girls), perhaps because strabismus is considered a barrier to ‘arranged marriage’ in traditional Indian society. The authors also emphasize the importance of maternal role in the upbringing of children in the Indian society by asking the mothers preferentially (and not the fathers) to answer the questionnaires. Did they believe that the fathers would be less distressed by the ‘problem’ than more ‘caring’ mothers? As a father, I find this too a gender bias on the part of the authors! Perhaps the extreme social groups are under-represented, as they may not be accessing care at all, even if it is nominally free.

The authors correctly emphasize that strabismus surgery is not just cosmetic, but has a vision-enhancing role. I would like to congratulate the authors on this important study that presents the care recipients' perspective in eye care. Many more such studies are needed in the Indian settings.

## References

[1] Kothari M, Balankhe S, Gawade R, Toshnival S (2009). Comparision of psycosocial and emotional consequences of childhood strabismus on the families from rural and urban India. Indian J Ophthalmol.

[2] O'Neal TD, Rosenbaum AL, Stathacopoulous RA (1995). Distance stereoacuity improvement in intermittent exotropic patients following strabismus surgery. J Pediatr Ophthalmol Strabismus.

[3] Archer SM, Musch DC, Wren PA, Guire KE, Del Monte MA (2005). Social and emotional impact of strabismus surgery on quality of life in children. J AAPOS.

[4] Lewallen S, Courtright P (2002). gender and use of cataract surgical services in developing countries. Bull World Health Organ.

